# Genetical and functional investigation of *fliC *genes encoding flagellar serotype H4 in wildtype strains of *Escherichia coli *and in a laboratory *E. coli *K-12 strain expressing flagellar antigen type H48

**DOI:** 10.1186/1471-2180-5-4

**Published:** 2005-01-24

**Authors:** Lothar Beutin, Eckhard Strauch, Sonja Zimmermann, Stefan Kaulfuss, Christoph Schaudinn, Andrea Männel, Hans R Gelderblom

**Affiliations:** 1 Department of Biological Safety, Robert Koch-Institut, Berlin, D-13353, Germany

## Abstract

**Background:**

Serotyping of O-(lipopolysaccharide) and H-(flagellar) antigens is a wideley used method for identification of pathogenic strains and clones of *Escherichia coli*. At present, 176 O- and 53 H-antigens are described for *E. coli *which occur in different combinations in the strains. The flagellar antigen H4 is widely present in *E. coli *strains of different O-serotypes and pathotypes and we have investigated the genetic relationship between H4 encoding *fliC *genes by PCR, nucleotide sequencing and expression studies.

**Results:**

The complete nucleotide sequence of *fliC *genes present in *E. coli *reference strains U9-41 (O2:K1:H4) and P12b (O15:H17) was determined and both were found 99.3% (1043 of 1050 nucleotides) identical in their coding sequence. A PCR/RFLP protocol was developed for typing of *fliC*-H4 strains and 88 *E. coli *strains reacting with H4 antiserum were investigated. Nucleotide sequencing of complete *fliC *genes of six *E. coli *strains which were selected based on serum agglutination titers, *fliC*-PCR genotyping and reference data revealed 96.6 to 100% identity on the amino acid level. The functional expression of flagellin encoded by *fliC*-H4 from strain U9-41 and from our strain P12b which is an H4 expressing variant type was investigated in the *E. coli *K-12 strain JM109 which encodes flagellar type H48. The *fliC *recombinant plasmid carrying JM109 strains reacted with both H4 and H48 specific antisera whereas JM109 reacted only with the H48 antiserum. By immunoelectron microscopy, we could show that the flagella made by the *fliC*-H4 recombinant plasmid carrying strain are constituted of H48 and H4 flagellins which are co-assembled into functional flagella.

**Conclusion:**

The flagellar serotype H4 is encoded by closely related *fliC *genes present in serologically different types of *E. coli *strainswhich were isolated at different time periods and geographical locations. Our expression studies show for the first time, that flagellins of different molecular weigth are functionally expressed and coassembled in the same flagellar filament in *E. coli*.

## Background

Bacterial strains belonging to the *Enterobacteriaceae *species *Escherichia coli *are common as commensals in the intestinal flora of humans and warm-blooded animals [1]. Typing systems for identification of related *E. coli *strains were developed in the early 1940ies when it became evident that certain *E. coli *strains were agents of infantile gastroenteritis [2]. In 1944, Kauffmann established the method of serological typing for *E. coli *O- (lipopolysaccharide) and H-(flagellar) antigens which allowed the grouping of *E. coli *strains according to their O:H-types (serotypes) [3]. Serotyping proved to be widely useful for identification of enteropathogenic *E. coli *(EPEC) strains from stools of diarrhoeic infants [4,5] and is successfully employed for characterization of pathogenic *E. coli *strains from both humans and animals [2,3].

The genetic analysis of *E. coli *populations by multilocus enzyme electrophoresis (MLEE) and multilocus sequence typing (MLST) allowed the detection of clonal types of strains which carry specific virulence markers and are associated with disease in humans [6,7]. It was shown that the O:H serotype is a good marker for identification of strains belonging to clonal types of pathogenic *E. coli *[6,7]. At present, 176 O- and 53 H-antigens are described for *E. coli *which can occur in different combinations in wildtype isolates of strains [2,3,5,8]. However, the large number of O- and H-antisera which are needed for *E. coli *serotyping and the laborious typing procedure restricts its usage to a few reference laboratories. Therefore, alternative typing methods were developed including molecular characterization of genes coding for the O- and H-antigens in *E. coli *[9-12]. Typing of *fliC *genes by PCR was successfully employed for characterization of human pathogenic O157:H7 and O26:H11 strains [13,14]. Analysis of the nucleotide sequence of *fliC *genes coding for flagellar antigens H7 and H6 revealed large sequence similarities between strains sharing the same H-type but different O-types [15,16]. Moreover, molecular typing of the *fliC *gene allows H-typing of non-flagellated (non-motile) *E. coli *isolates which cannot be analyzed for their flagella with H-specific antisera [14,15,17].

The flagellar type H4 is frequently occurring in *E. coli *belonging to many different O-groups including strains of shiga toxin-producing *E. coli *(STEC) and extraintestinal pathogenic serotypes [18-20], (K.A. Bettelheim, The VTEC table, May 2003, ). Moreover, a cryptic *fliC*-H4 gene was described to be present and to be spontaneously expressed in *E. coli *strain P12b (O15:H17) which carries another type of flagella called H17 which is not encoded by the *fliC *gene [21,22]. Therefore, we became interested in the genetic variability of flagellar H4 genes in *E. coli *strains belonging to different O-serogroups and pathotypes. We have used the published nucleotide sequence of the *fliC *gene present in the *E. coli *H4 reference strain U9-41 (accession AB028472) to develop a PCR which allows discrimination between the *fliC*-H4 gene variants. Nucleotide sequence analysis of the *fliC *gene was performed on other *E. coli *H4 strains that either showed deviations in the PCR analysis or were reported to harbour allelic types of the H4-*fliC *gene [9] or revealed differences in the agglutination reaction compared to the reference strains. To study the expression of different flagellar H4 types we have cloned their corresponding *fliC *genes and have introduced them into the laboratory *E. coli *K-12 strain JM109 [23]. Expression of recombinant flagella was demonstrated by serotyping and by immuno electron microscopy.

## Results

### Serological detection of the flagellar H4 antigen in different *E. coli *wildtype host strains

*E. coli *reference strains U9-41 (O2:K1:H4) and P12b (O15:H17) [3] and the *E. coli *K-12 strain JM109 (O-rough:H48) (this study), which was used for expression studies were investigated for inhibition of motility in swarm-agar containing 1:600 dilutions of either H4, or H48-antiserum (see Methods). U9-41 and P12b were fully inhibited for their motility in the presence of H4 antiserum derived from strains U9-41 or from P12b but not in the presence of H48 specific antiserum. The *E. coli *K-12 strain JM109 was not inhibited for motility by H4 antisera but by H48 antiserum. These findings indicate that H4 antisera specifically inhibited the motility of *E. coli *H4 strains and that the antigenically different flagellar type H17 was not expressed or lost in our P12b isolate, similar as previously described with spontaneously arising variants of P12b [24]. We became interested if other *E. coli *H17 strains would also carry the genes for expression of H4 type flagella as it was described for P12b [21,22,24]. For this, we have investigated five additional *E. coli *H17 strains (872-69, 107-74, 305-78, 870-69 and 327-01) for their serological reaction with different H4 specific antisera and all strains showed specific positive reactions (Table 1). H-serotyping performed with strains from the collection of the Robert Koch-Institute revealed 88 *E. coli *strains which agglutinated with H4 antisera and the results obtained with 10 representative strains are shown in Table 1. All strains agglutinated with both, H4_U9-41 _and H4_P12b _antiserum, but were not agglutinating with antisera made against other H-antigens (data not shown). Differences in agglutinating titers between H4_U9-41 _and H4_P12b _antisera were not more than twofold with either strain indicating that both sera were similar for their specificity (Table 1). The strains P7d, E1541-68, 107-74 and 305-78 showed significantly lower agglutination titers with H4 antisera than did the reference strains U9-41 and P12b, which had been used for production of H4 typing sera, respectively (Table 1). These findings prompted us to compare all the H4 strains with all other *E. coli *H-types for polymorphisms in the *fliC *gene by HhaI digestion of *fliC*-PCR products as described in the Method section.

### HhaI-RFLP typing of *fliC *genes in *E. coli*

**Figure 1:**
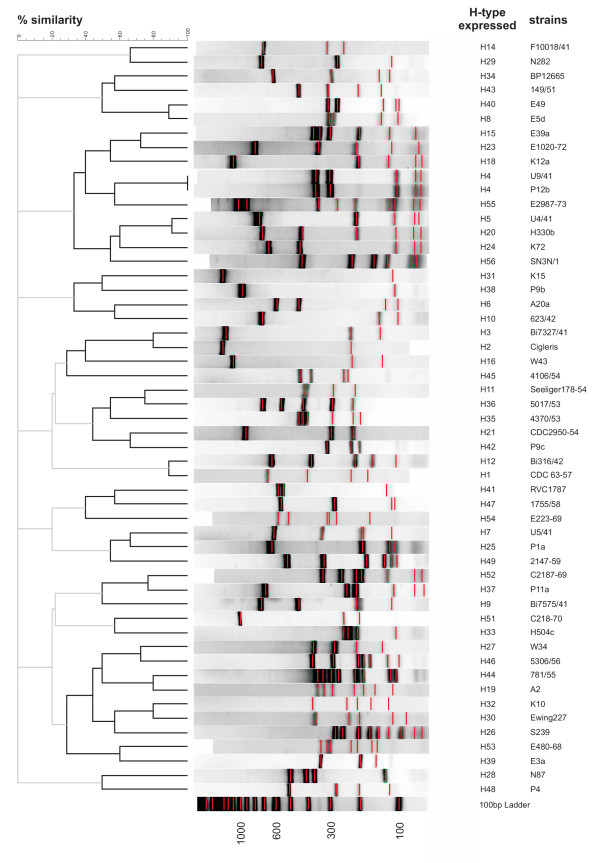
HhaI digested *fliC *PCR products obtained with primers fliC-1 and fliC-2 from *E. coli *reference strains for 53 different expressed H-types as indicated at the right side. Similarity of restriction fragment patterns was calculated with BioNumerics software and is indicated by the dendrogram on the left side. The flagellar antigens of strains encoding H-types H3, H17, H35, H36, H44, H47, H53, H54 and H55 are not encoded by *fliC *but by other genes (*flkA*, *fllA, flmA *and others) in the corresponding *E. coli *strains and the *fliC *HhaI patterns obtained from these strains do therefore not correspond to their H-serotypes [21, 25, 26, 27].

PCR products obtained with primers fliC-1 and fliC-2 were digested with HhaI to obtain H-serotype specific RFLP patterns from reference strains for the 53 different *E. coli *H-serotypes [3,9]. HhaI-RFLP typing of *E. coli fliC *genes coding for flagellar types H1 to H56 revealed individual patterns corresponding to the different H-serotypes (Fig. 1). Flagellar antigens H3, H17, H35, H36, H44, H47, H53, H54 and H55 were reported to be not encoded by *fliC *but by other genes (*flkA*, *fllA, flmA *and others) in the corresponding *E. coli *strains and the *fliC *HhaI patterns obtained from these strains do therefore not correspond to their H-serotypes [21,25-27]. HhaI-RFLP patterns were found conserved among strains sharing the same H-serotype independent of their O-antigen as previously described [9] (data not shown). An exception was found for H-serotypes 2, 8, 18, 19, 21, 33 and 47 in which single strains possessed different HhaI-RFLP patterns when compared with the corresponding H-type reference strain (data not shown). The HhaI-RFLP patterns of different H-types were distinguishable from each other (Fig. 1). The results from HhaI-RFLP typing corresponded with the earlier reports showing that the *fliC *gene in strain P12b codes for flagella of serotype H4. The relationship between *fliC *genes present in the different *E. coli *strains was further investigated by nucleotide sequencing.

### Nucleotide sequence analysis of the *fliC *genes present in representative *E. coli *H4 strains

**Figure 2:**
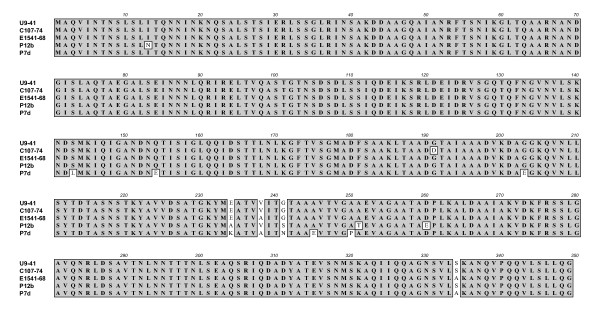
Alignment of the deduced FliC (flagellin) sequences from *E. coli *strains representing the flagellar antigen H4 and its genetic variants: U9-41 (accession BAA85081); C107-54 (accession CAE53943), E1541-68 (accession CAD60547); P12b (accession CAD56695); and P7d (accession CAE53942).

To obtain the entire coding region of the *fliC *gene in *E. coli *strains oligonucleotide primers fliC-5 and fliC-6 were deduced from the *fliC*-H4 chromosomal region and applied to amplify the corresponding chromosomal regions from *E. coli *strains U9-41 (O2:K1:H4), P12b (O15:H17), U1-41 (O5:K4:H4), P7d (O68:H4), C107-74 (O15:H17) and E1541-68 (O154:H4) as listed in Table 1. These strains were taken as flagellar type H4 representatives according to previously published results [3,9] and according to the H-serotyping performed in this study. The analysis of the coding sequences of the *fliC *genes present in these strains revealed in all cases a length of 1050 bp. As a control, the *fliC *gene of the strain U9-41 was sequenced and found to be identical to the previously deposited *fliC *sequence (accession AB028472). The identity of the *fliC*-H4 sequences ranges from 97,6 % to 100 %. The greatest sequence difference to the *fliC *gene coding sequence of strain U9-41 was found in the *fliC *gene of strain P7d (exchange of 20 nucleotides), whereas the *fliC *sequences of strains U1-41 and U9-41 were identical to each other. All deduced flagellins have a length of 349 amino acids. The greatest deviation in the primary structure to the FliC protein of the reference strain U9-41 was again observed for the FliC protein of strain P7d (exchange of 9 aa in a stretch of 349 aa). Fig. 2 shows the alignment of the deduced FliC proteins of all investigated strains. The FliC protein of strain U1-41 is 100% identical to that of strain U9-41 and therefore not shown. Similarities of the deduced amino acid sequences of the FliC proteins (flagellins) are summarized in Table 2.

### PCR based detection of *fliC*-H4 specific DNA sequences

**Figure 3:**
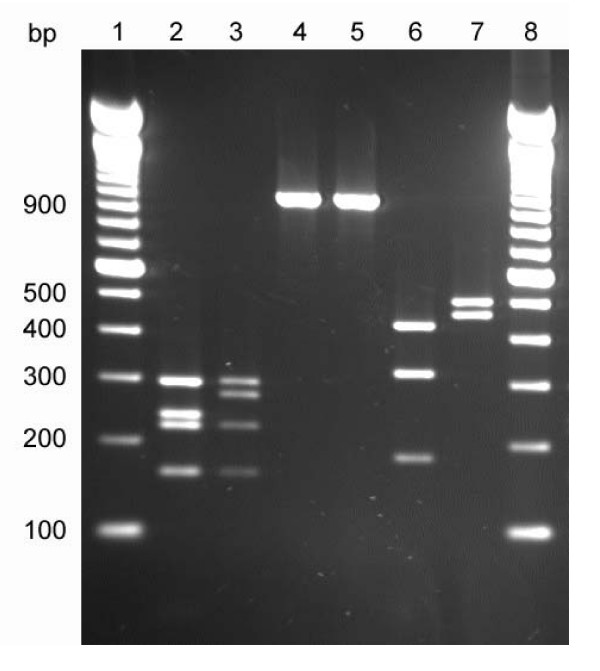
Electrophoretic separation of restriction enzyme digested *fliC *PCR products (primers fliC-1 and fliC-2) of *E. coli *strains U9-41 and P12b on 2% agarose: Lanes: 1+8= molecular weight standard; 2 = U9-41 (HpaII); 3 = P12b (HpaII); 4 = U9-41 (undigested); 5 = P12b (undigested); 6 = U9-41 (MboI); 7 = P12b (MboI). Sizes of restriction fragments are listed in Table 2.

Amplification of the *fliC *genes present in *E. coli *strains U9-41 and P12b with primers fliC-1 and fliC-2 resulted in the generation of a 953 bp internal PCR product with both strains (Fig. 3, lanes 4+5). The nucleotide sequences of this stretch of DNA of strains U9-41 and P12b were compared for restriction enzymes which cut at different sites in the *fliC*-H4_U9-41 _and *fliC*-H4_P12b _sequence. HhaI was found to cut at identical sites confirming the results from HhaI RFLP typing (Table 3). In contrast, enzymes HpaII and MboI were found to generate each different restriction fragments from PCR products of the *fliC*-H4_U9-41 _and *fliC*-H4_P12b _gene, respectively. Both enzymes were taken for RFLP typing of amplified *fliC *genes (primers fliC-1 and fliC-2) from 88 *E. coli *strains which showed agglutination reactions with H4 antisera (Table 3). Eighty-six of the 88 strains showed HpaII and *Mbo*I restriction profiles which corresponded to the patterns obtained with strain U9-41 (O2:K1:H4) (Table 3 and Fig. 3). Exceptions were made by strains P7d (O68:H4) and P12b (O15:H17) which showed individual restriction patterns which differed from all other H4 strains investigated in this study. (Table 3).

### Cloning and expression of *fliC*-H4 genes in the *E. coli *K-12 strain JM109

**Figure 4:**
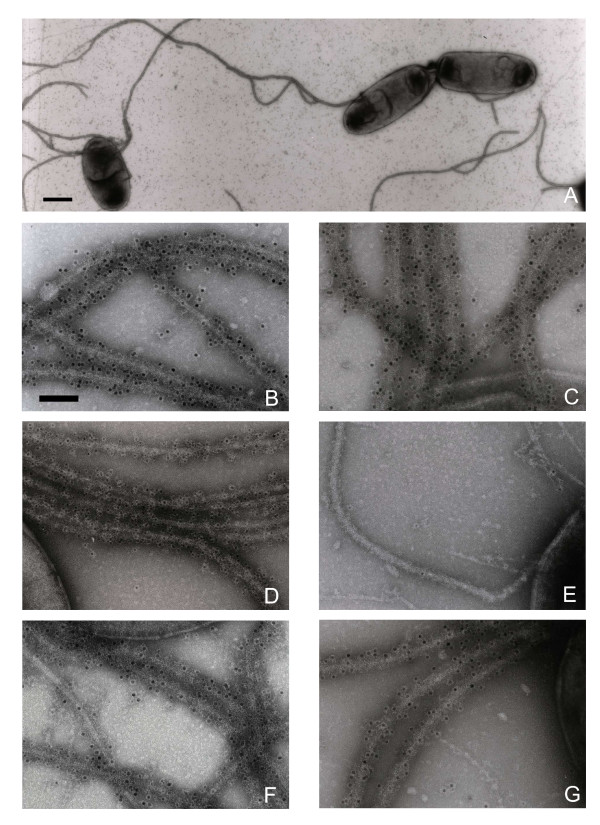
(A) Low power micrograph of *E. coli *strain TPE1978 cells showing the density of the bacterial samples used for indirect IEM and the presentation of flagella (bar length = 1 μm) (B) IEM of strain TPE1978 flagella after incubation with rabbit flagellar H48 antiserum (1:1000) and detection of bound antibody by anti-rabbit-IgG- 10 nm gold (1:20), bar length = 100 nm. (C) Strain JM109 flagella after incubation with rabbit flagellar H48 antiserum and detection of bound antibody by anti-rabbit-IgG- 10 nm gold. (D) Strain TPE1978 flagella after incubation with rabbit flagellar H4 antiserum (1:1000) and detection of bound antibody by anti-rabbit-IgG- 5 nm gold. (E) Strain JM109 flagella after incubation with rabbit flagellar H4 antiserum and detection of bound antibody by anti-rabbit-IgG- 5 nm gold. (F) Double-labeling IEM of strain TPE1978 after sequential incubations with rabbit flagellar H4 antiserum and anti-rabbit-IgG 5 nm gold, followed by rabbit flagellar H48 antiserum detected by anti-rabbit-IgG- 10 nm gold. Both, 5 nm and 10 nm gold markers are bound at comparable amounts over all flagella present on the bacteria. (G) Double-labeling IEM of strain JM109 after sequential incubations with rabbit flagellar H4 antiserum and anti-rabbit-IgG- 5 nm gold followed by rabbit flagellar H48 antiserum and anti-rabbit-IgG- 10 nm gold. Only H48 specific (10 nm) gold particles are bound to the flagella of JM109.

In order to study the functional expression of the *fliC*-H4 genes in a different genetic background we cloned the corresponding PCR products of strains U9-41 and P12b into the vector pLITMUS38 as described in the Methods section. The *fliC *coding regions were inserted downstream of the *lacZ *promoter of pLITMUS38 and the *fliC *recombinant plasmids were transformed into the laboratory *E. coli *K-12 strain JM109 [23]. JM109 was serotyped as O-rough:H48, and it showed the same HhaI-RFLP *fliC*-pattern as the *E. coli *reference strain P4 (O16:H48) [3] (Fig. 1). The functional expression of the cloned *fliC*-H4 genes in the JM109 derivative strains TPE1976 (*fliC*-H4_U9-41 _clone) and TPE1978 (*fliC*-H4_P12b _clone) was analyzed by tube agglutination with H4 and H48 antisera, respectively. The strains TPE1976 and TPE1978 showed agglutination with H48 and H4 sera whereas the parental JM109 strain reacted only with H48 serum (Table 4). To find out if the flagellins of the *fliC *recombinant plasmid carrying JM109 strains were co-assembled in all flagella or assembled separately we studied the parental strain JM109 and its *fliC-*H4 derivative TPE1978 by IEM (Fig. 4B–G). Both strains were found to express 2-4 flagella per cell which appeared morphologically typical as long helical filaments (diameter 18 nm, length up to 20 μm) (Fig. 4A), [29]. The reaction of H48 antiserum with bacteria followed by detection of adsorbed antibodies with anti-rabbit-IgG coupled with 10 nm immuno-gold particles showed a specific and homogeneous labelling of flagella present on the surfaces of JM109 (Fig. 4C) and of TPE1978 (Fig. 4B). When H4 serum was used, only flagella of strain TPE1978 became immuno-labelled (5 nm gold particles) (Fig. 4D) and no labeling was observed with JM109 (Fig. 4E), confirming H-serotyping results. Sequential double labeling experiments with H48 and H4 antibodies resulted in staining of all flagella on the surfaces of JM109 (Fig. 4G) and TPE1978 (Fig. 4F). However, both 5 nm (H4 label) and 10 nm (H48 label) gold particles were only bound to flagella of TPE1978 (Fig. 4F) whereas the flagella of JM109 were exclusively labelled with 10 nm gold particles which indicates the H48 antigen (Fig. 4G). These results demonstrate that both H48 and H4 flagellins are co-assembled in the flagella made by the *fliC*-H48/H4 genes in strain TPE1978.

## Discussion

The *fliC *genes of representative *E. coli *strains for the 53 different H-types were recently investigated and compared for their nucleotide sequences [21]. Among the H-type reference strains which were not analyzed for their complete *fliC *gene sequences, were the reference strains for the *E. coli *H4 (U9-41) and H17 (P12b) flagellar antigen [21,22]. In this work, we performed a complete characterization of the *fliC-*H4 genes from different *E. coli *strains with primers which were generated on the basis of the previously published *fliC *sequence (accession AB028472) of the *E. coli *H4 reference strain U9-41. The comparison of the *fliC *gene sequence encoding H4 flagella in strain P12b with the *fliC *sequences of five other *E. coli *H4 or H17 strains revealed a high similarity on the DNA and on the amino acid level (Fig. 2 and Table 2). We established a PCR/RFLP typing assay for genotypic investigation of clinical *E. coli *isolates which reacted with H4 antisera. Genotyping of 88 *E. coli *strains comprising 20 different O-serogroups (Table 3) revealed that 86 of the strains gave RFLP patterns with HhaI, MboI and HpaII which were indistiguishable from the prototype *fli*C-H4 gene and only two strains showed alternative patterns. The detection of some genetic variants in the *fliC*-H4 gene of *E. coli *strains studied here points to a sequence diversity similar as described previously for the *fliC *genes of *E. coli *H6 and H7 strains [15,16]. These results correspond to previous findings indicating that the H4 antigens in different *E. coli *typing strains are not fully identical [20,24,28].

It was suggested earlier that the strain P12b encodes two flagellins, H4 and H17, which are subject to phase variation for their expression [22] and mutants of P12b could be isolated which expressed only the H4 antigen [24]. Recent studies have demonstrated that the *fliC *gene of strain P12b encodes flagellar type H4 and it was suggested that the gene for the H17 flagellin is encoded by a locus outside *fliC*, however the gene responsible for the flagellar type H17 was not identified in the study [21]. In our study, cultures of strain P12b were fully inhibited for motility and swarming in the presence of H4 antiserum but not in the presence of H48 antiserum which was used a non-specific control. This result indicates that H17 type flagella were not expressed or lost from our P12b isolate, similar as previously described with mutant strains of P12b [24].

The functional expression of the cloned *fliC*-H4_U9-41_and *fliC*-H4_P12b _genes in the genetic background of *E. coli *K-12 strain JM109 which shows flagellar serotype H48 confirmed their coding capacity. Since we found coexpression of cloned flagellins with the parental H48 flagellin, we became interested in the composition of flagella made by the *fliC *recombinant plasmid carrying JM109 strains. Electron microscopy of flagella from JM109 and from the *fliC*-H4_P12b _recombinant plasmid carrying strain revealed no differences between JM109 and TPE1978 showing both a typical helical organization of normal sized flagella on their surface (Fig. 4). Immuno-gold staining of bacteria which were prior incubated with H48 and H4 antisera revealed high specificity of the rabbit antisera for the flagellar structure (Fig. 4B–G). Single (Fig. 4 B+D) and double labelling (Fig. 4 F) experiments with different sized gold markers demonstrated that all flagella present on the surface of TPE1978 were labelled after incubation with H48 and H4 antiserum. Our results indicate that both H48 and H4 flagellins are coassembled in the flagella made by the *fliC*-H4_P12b _recombinant plasmid carrying strain. This finding is surprising in view of the molecular weight size differences found between H48 and H4 flagellin. To our knowledge, the assembling of two different flagellins in the same filament has not yet been demonstrated before. The H48 flagellin of *E. coli *K-12 (accession AE000285) is a 51.3 kDa protein consisting of 498 amino acids and thus much larger than the 36.3 kDa H4 flagellin which is composed of 349 amino acids. However, both flagellins share conserved N- and C-terminal sequences which are known to be involved in the structural assembly of flagella [29]. H48 and H4 flagellins differ largely for their central regions which are not involved in flagellar assembly and function but which contain flagellar antigenic epitopes [29].

We were able to show that the introduction of an isolated *fliC *gene in *E. coli *can change the antigenic properties of the flagella made by this strain. Horizontal transfer of *fliC *genes may contribute to the diversity of flagellar serotypes by recombination within *E. coli *recipient strains. The mammalian host immune system is the driving force for continuous selection of new flagellar antigens in *E. coli*. Published data indicate that both mutation and recombination events in the *fliC *gene have taken place in the evolution of *E. coli *flagellar antigens [15,16].

## Conclusions

Our *fliC *sequence data have shown that the flagellar type H4 which is present in *E. coli *strains of clinical importance covers several genetic variants which are closely related to each other. We have shown for the first time that flagellins of different molecular size are expressed and coassembled into functional flagella in a laboratory *E. coli *K-12 strain.

## Methods

### Bacterial strains

The reference strains used for production of antisera for O- and H-typing of *E. coli *were obtained from the International Escherichia and Klebsiella Centre, Statens Seruminstitut, Copenhagen, Denmark and are described elsewhere [3]. Origin and serotype data on five additional strains with flagellar type H17 are listed in Table 1. A laboratory collection of 88 *E. coli *isolates originating from humans and animals was investigated by PCR/RFLP typing for *fliC*-H4 genes. These strains were previously investigated for the O-types and for production of Shiga-toxins (Stx) and were isolated in different countries between 1941 to 2002 [3,19,30] (Table 3). The *E. coli *K-12 strain JM109 is described elsewhere [23].

### Production of *E. coli *O and H-specific antisera

Rabbit antisera against the different O- and H-antigens of *E. coli *were prepared according to ∅rskov and ∅rskov [3]. Antisera for typing of flagellar antigens H4 were produced with reference strains U9-41 (O2:K1:H4) and P12b (O15:H17) [3]. Our strain P12b was found to express its *fliC *encoded H4 antigen and produced flagellar type H4 (this work).

### Motility inhibition test

Expression of flagella and swarming of *E. coli *strains was tested by inoculating bacteria in tubes containing 10 ml portions of swarm-agar (L-broth + 0.3% agar) as described [3]. Inhibition of motility of *E. coli *strains in the presence of flagellar-specific antiserum (H4 and H48) was tested in swarm-agar containing a 1:600 dilution of the respective antiserum. Cultures which were inhibited for motility were observed over two weeks for possible switch to motility by phase variation.

### Serological typing of H-antigens of *E. coli*

H-serotyping was performed as described [3]. In brief, bacteria were grown in tubes containing 10 ml 0.3% semi-solid LB-agar [23] for two to three passages. Highly motile bacteria were transferred in LB-medium, incubated 6h at 37°C and inactivated by addition of 0.5% formaldehyde in solution. Agglutination reactions were performed in two fold dilutions of 0.5 ml portions of serum in phosphate-buffered saline pH 7.4 (PBS) [22] with 0.5 ml formalized bacteria in glass tubes which were incubated for 2h at 50°C. Agglutination tests were read by eye immediately after incubation as described [3].

### PCR-typing of *fliC* genes

The oligonucleotide primers fliC-1 (5' CAA GTC ATT AAT AC(A/C) AAC AGC C 3') and fliC-2 (5' GAC AT(A/G) TT(A/G) GA(G/A/C) ACT TC(G/C) GT 3') were used for amplification of internal parts of *fliC *genes present in the *E. coli *reference strains as described [9]. The PCR was performed for 25 cycles at 94°C for 60 sec, 55°C for 60 sec and 72°C for 120 sec [9]. PCR products of sizes varying between 950 to 2500 bp were obtained with *E. coli *reference strains for 53 different H-types [3] (Figure 1). Amplified DNA was digested with HhaI and the resulting restriction fragments were compared on 2% agarose gels. Restriction enzymes HpaII and MboI were used for characterization of fliC-H4 specific PCR products. Gel images were stored digitally and analyzed with BioNumerics software, version 2.5 (Applied Maths, Kortrijk, Belgium) for similarity (Dice, complete linkage) (Fig. 1).

### Nucleotide sequence analysis of *fliC *genes

Two primers deduced from the published *fliC*-H4 sequence (AB028472) were used for the amplification of the entire *fliC*-H4 coding regions. The PCR was performed for 30 cycles: 30 sec at 94°C, 60 sec at 58.1°C and 90 sec at 72°C with primers fliC-5 (5'-TGA GTG ACC AGA CGA TAA CAG GG-3') and fliC-6 (5'-GGA CGA TTA GTG GGT GAA ATG AGG-3') and yielded a 1243 bp product. PCR products were purified with the QIAquick™ PCR Purification Kit (Qiagen, Hilden, Germany) and used for sequencing. Sequencing reactions were carried out using the dye terminator chemistry (PE Applied Biosystems, Darmstadt, Germany) and separated on an automated DNA sequencer (ABI PRISM^® ^3100 Genetic Analyzer). The sequences were analysed using the Lasergene software (DNASTAR, Madison,WI, USA) and the Mac Vector software (Oxford Molecular Group, Campell, CA, USA) to assemblings and alignings.

### Nucleotide sequence accession numbers

The nucleotide sequence of the genomic region of *E. coli *strain P12b (O15:H17) with a size of 1234 bp containing the *fliC *gene for flagellin has been submitted to EMBL data library under accession number AJ515904. The coding sequences of the different *fliC *genes from the following strains have been assigned the following accession numbers: AJ605764 for strain U1-41 (O5:K4:H4), AJ605765 for strain P7d (O68:H4), AJ605766 for strain C107-74 (O15:H17) and AJ536600 for strain E1541-68 (O154:H4). The origin of the strains is listed in Table 1.

### Molecular cloning of *fliC *gene PCR products

PCR products encompassing the complete coding sequences of the *fliC*-H4 genes were obtained from genomic DNA prepared as described [9] of *E. coli *strains U9-41 and P12b using primers fliC-5 and fliC-6. The amplification products were inserted into the vector pLITMUS38 (New England Biolabs, Beverly, MA, USA) digested with EcoRV. The orientation of the the insert PCR products was determined by using commercially available sequencing primers LITMUS forward 28/38 and LITMUS Reverse 28/38 (New England Biolabs).

### Immuno electron microscopy (IEM) of *E. coli *flagellar antigens

Motile cultures of *E. coli *strains were produced by repeated passage on semi-solid agar followed by growth in L-Broth as described above. Cultures carrying recombinant pLITMUS38 plasmids were grown in the presence of 100 μg/ml ampicillin. IEM was performed using fresh, non-formalized cultures of motile bacteria. For IEM, aliquots of respective bacterial cultures were diluted 1:2 in PBS pH 7.2 and adsorbed onto glow-discharge treated 400 mesh grids coated with Pioloform and carbon (Wacker Chemie, Munich, Germany) [31]. Grids with adsorbed bacteria were preincubated for 30 min at room-temperature with blocking buffer (0.1 % bovine serum albumine (Sigma, Deisenhofen, Germany) in PBS). Rabbit anti-H48- and anti-H4 hyperimmune sera were diluted 1:1000 in blocking buffer. After conditioning, specimens were incubated for 30 min at room temperature on droplets of the specific, unlabelled antibodies. Non-bound antibody was removed by washing the grids twice for 10 min on blocking buffer. Immuno-specifically bound primary antibodies were detected using anti-rabbit-IgG-gold 5 or -gold 10 nm conjugates (British Bio Cell International Ltd, Cardiff, UK). The conjugates were diluted 1:20 in blocking buffer and reacted for 30 min at room temperature. Unbound conjugate was removed by a sequence of washing steps (two times with blocking buffer for 5 min each; once with PBS for 3 min and a final wash with double destilled water for 3 min) at room temperature. Before negative staining with 1 % uranyl acetate (pH 4.0–4.5), the grids were washed rapidly on 4 droplets of double destilled water. The preparations were analyzed with an EM 10 electron microscope (Zeiss-LEO, Oberkochen, Germany) at an accelerating voltage of 80 kV. To look for different antigenic determinants expressed on the flagella of strains JM109 or TPE1978, double immuno-labelling was performed using H48 and H4 antisera sequentially and two anti-rabbit-IgG-gold conjugates with different sized markers for the detection of the bound primary unlabelled rabbit antibody. Both *E. coli *strains were incubated with rabbit anti-H4 (1:1000) followed by anti-rabbit-IgG-5 nm gold and two washing steps on droplets of blocking buffer for 10 min. The samples were subsequently incubated with anti H48 antibody (1:1000) followed by incubation with anti-rabbit-IgG- 10 nm gold. Removal of surplus conjugate and negative staining were performed as detailed above.

## Author's contribution

LB conceived of the study and carried out PCR genotyping and coexpression studies. ES carried out sequence determination and alignments and construction of recombinant plasmids. SZ and SK performed serological assays and PCR genotyping. CS performed analysis of *fliC *recombinant plasmid carrying strains. AM and HG developed the IEM methodology and HG contributed to the data analysis. All authors participated in review and preparation of the final manuscript.

## Figures and Tables

**Table 1:** Agglutination titers with H4 antisera derived from strains U9-41 and P12b

Strain	reported serotype	agglutination with antisera^a^	
		
		H4_U9-41_	H4_P12b_
U9-41	O2:K1:H4^b^	12800	12800
U1-41	O5:K4:H4^b^	25600	25600
P7d	O68:H4^b^	1600	3200
E1541-68	O154:H4^b^	3200	1600
P12b	O15:H17^c,e^	6400	12800
872-69	O20:H17^d,e^	12800	12800
107-74	O15:H17^d,e^	3200	3200
305-78	O15:H17^d,e^	3200	3200
870-69	O20:H17^d,e^	6400	12800
327-01	O77:H17^d,e^	12800	12800

a) reciprocal value of agglutination titers with antisera (the same results were obtained in two separate experiments). H4_U9-41 _and H4_P12b _indicates the H4 antisera produced with strains U9-41 and P12b, respectively.

b) *E. coli *standard test strain [3-5]

c) *E. coli *standard test H-test strain [3-5], able to produce spontaneously a variant expressing flagellar antigen H4 and containing a *fliC*-H4 gene [21, 22]

d) strains and serotype data provided from Flemming Scheutz, Statens Seruminstitut, Copenhagen, Denmark

e) all H17 strains were shown to carry a *fliC*-H4 genotype and expressed flagellar H4 antigens (this work).

**Table 2:** Amino acid identitity/divergence between the deduced FliC proteins of the six investigated *E. coli *strains (aligned length 349 aa, no gaps).

		percent identity					
		U9-41	U1-41	C107-74	E1541-68	P12b	P 7d

Percent divergence	U9-41	***	100.0	99.7	99.4	98.6	97.4
	U1-41	0.0	***	99.7	99.4	98.6	97.4
	C107-74	0.3	0.3	***	99.1	98.3	97.1
	E1541-68	0.6	0.6	0.9	***	98.0	98.0
	P12b	1.4	1.4	1.7	2.0	***	96.6
	P 7d	2.6	2.6	2.9	2.0	3.5	***

**Table 3:** PCR/RFLP typing of *fliC *genes in *E. coli *H4 strains with restriction enzymes HhaI, HpaII and MboI

	DNA fragments (bp) obtained by enzymatic digestion of the 953 bp PCR product obtained with primers fliC-1 and fliC-2		
*fliC*-genotype (prototype strain)	HhaI	HpaII	MboI

*fliC*-H4 (U9-41)^a^	362, 304, 104, 66, 50, 29^d^, 20^d^, 16^d ^2^d^	296, 240, 225, 159, 33^d^	429, 313, 182, 29^d^
*fliC*-H4 (P7d)^b^	362, 304, 104, 66, 50, 29^d^, 20^d^, 16^d^,2^d^	296, 273, 225, 159	429, 313, 182, 29^d^
*fliC*-H4 (P12b)^c^	362, 304, 104, 66, 50, 29^d^, 20^d^, 16^d^,2^d^	296, 273, 225, 159	495, 458

a) PCR/RFLP patterns found in strains U9-41, U1-41, E1541-68, C107-74 and in 82 *E. coli *strains which reacted with H4 antisera. The serotypes of all 86 strains are: O2:K1 (4 strains), O5 (2), O7 (1), O8 (1), O13 (1), O15 (2), O20 (2), O50 (1), O60 (1), O77 (1), O78 (1), O99 (3), O111 (9), O113 (32), O114 (5), O117 (1), O119 (1), O141 (3), O154 (1), O176 (1), O181 (1), O-untypable (9), O-rough (3). Forty-two of the strains belonging to O-groups O60, O113, O114, O141 as well as O-rough and O-untypable strains produced Shiga-toxins.

b) RFLP patterns found with strain P7d (O68:H4)

c) RFLP-patterns found with strain P12b (O15:H17)

d) fragments smaller than 50 bp are not detectable on 2% agarose gels (Fig. 3). Exact fragment sizes were calculated on the basis of nucleotide sequence analysis for each of the restriction enzymes used

**Table 4:** H-Agglutination reaction of *fliC*-H4 recombinant plasmid carrying *E. coli *K-12 strains

Strain	serotype or *fliC *recombinant JM109 derivative	agglutination with H-specific antisera^a^		
		
		H48	H4_U9-41_	H4_P12b_
U9-41	O2:K1:H4	<200^b^	12800	12800
P12b	O15:H17	<200^b^	6400	12800
JM109^c^	O-rough:H48	12800	<200^b^	<200^b^
TPE1976	JM109 (pLITMUS38-*fliC*-H4_U9-41_)^d^	12800	12800	12800
TPE1978	JM109 (pLITMUS38-*fliC*-H4_P12b_)^e^	12800	6400	12800

a) reciprocal value agglutination titers with antisera (the same results were obtained in two separate experiments). H4_U9-41 _and H4_P12b _indicates the H4 antisera produced with strains U9-41 and P12b, respectively.

b) no agglutination with start serum dilution 1:200

c) *E. coli *K-12 [23]. d) *fliC*-H4 gene cloned from strain U9-41

e) *fliC*-H4 gene cloned from strain P12b
